# A Systematic Review of Butterfly Pea Flower (*Clitoria ternatea* L.): Extraction and Application as a Food Freshness pH-Indicator for Polymer-Based Intelligent Packaging

**DOI:** 10.3390/polym15112541

**Published:** 2023-05-31

**Authors:** Nur Nabilah Hasanah, Ezzat Mohamad Azman, Ashari Rozzamri, Nur Hanani Zainal Abedin, Mohammad Rashedi Ismail-Fitry

**Affiliations:** 1 Department of Food Technology, Faculty of Food Science and Technology, Universiti Putra Malaysia, UPM Serdang 43400, Selangor, Malaysia; nabilah3003@gmail.com (N.N.H.); ezzat@upm.edu.my (E.M.A.); rozzamri@upm.edu.my (A.R.); hanani@upm.edu.my (N.H.Z.A.); 2 Halal Products Research Institute, Universiti Putra Malaysia (UPM), Putra Infoport, UPM Serdang 43400, Selangor, Malaysia

**Keywords:** anthocyanin, *Clitoria ternatea* L., ultrasonic extraction, pH-responsive indicator, food freshness, intelligent packaging

## Abstract

The butterfly pea flower (*Clitoria ternatea* L.) (BPF) has a high anthocyanin content, which can be incorporated into polymer-based films to produce intelligent packaging for real-time food freshness indicators. The objective of this work was to systematically review the polymer characteristics used as BPF extract carriers and their application on various food products as intelligent packaging systems. This systematic review was developed based on scientific reports accessible on the databases provided by PSAS, UPM, and Google Scholar between 2010 and 2023. It covers the morphology, anthocyanin extraction, and applications of anthocyanin-rich colourants from butterfly pea flower (BPF) and as pH indicators in intelligent packaging systems. Probe ultrasonication extraction was successfully employed to extract a higher yield, which showed a 246.48% better extraction of anthocyanins from BPFs for food applications. In comparison to anthocyanins from other natural sources, BPFs have a major benefit in food packaging due to their unique colour spectrum throughout a wide range of pH values. Several studies reported that the immobilisation of BPF in different polymeric film matrixes could affect their physicochemical properties, but they could still effectively monitor the quality of perishable food in real-time. In conclusion, the development of intelligent films employing BPF’s anthocyanins is a potential strategy for the future of food packaging systems.

## 1. Introduction

Anthocyanins in *Clirotia ternatea* L. are present in the form of polyacrylate anthocyanins also known as Ternatins. They are among the stable forms of anthocyanin [1], and their stability is higher than the non-acylated ones. This plant is primarily found in tropical regions, where it needs intense sunlight and is immune to abiotic stress [2]. In the butterfly pea flower (BPF), the big advantage of polyacrylate anthocyanin is that it is known to be employed as a natural food dye [3]. The most noticeable feature of the BPF is its petal, which has a highly appealing blue colour [4]. In Malaysian dishes, BPFs are used to introduce blue colour to white rice, namely *nasi kerabu*. Other than that, a refreshing lemonade drink is also made using the BPF and is traditionally used in Southeast Asia as a herbal tea. Due to their unique colour properties, BPFs have been utilised for other applications in foods. On top of that, the significant colour-change properties of BPF are also one of the most important criteria for spoilage detection in food products which come from natural sources (anthocyanin) [5]. In this context, the use of intelligent pH-colourimetric packaging is an innovative system for distinguishing, monitoring, securing, and assuring food safety and quality [6].

The increase in consumer awareness of food safety opens a new area of research, with the incorporation of natural colourants from several plant-based sources being recommended as a beneficial alternative to toxic synthetic (chemical) dyes. Generally, consumers frequently use the shelf-life date (expiry date) displayed on the packaging to determine and assess the freshness and quality of perishable foods [7]. However, some perishable food products, such as muscle food, fresh fruits, and vegetables, cannot be assessed for their quality and freshness only based on their shelf-life date [8]. Thus, it has been discovered that polymer-based intelligent packaging can act as an indicator required for monitoring the spoilage of food in real-time and instilling confidence in consumers upon purchase.

According to Poh [8], colour transformations are due to the molecular structure of anthocyanins having an ionic nature. In acidic conditions (pH value < 2), the anthocyanins will appear red, which is the formation of flavylium ions. As the pH increases, the first deprotonation occurs and creates cations, converting flavylium ions into a neutral quinonoid base [9]. Apart from that, anthocyanins in BPFs have a blue colour property in neutral pH; the colour of anthocyanin will change to green-light yellow with an increasing pH condition. The changes occur due to tautomerisation which changes hemiketal structure to cis-chalcone and trans-chalcone as a result of the deprotonation from the C5-OH that has been set aside [10]. According to Rahim et al. [5], microbial and biochemical spoilage causes changes in food pH; thus, intelligent packaging made from polymer infused with active compounds such as anthocyanin can help identify the changes in food pH through a colour response due to reactions between the delphinidin of anthocyanin and volatile amines produced by bacteria and the enzymes [11]. Therefore, this review covers the morphology, anthocyanins extraction, and applications of BPF anthocyanin-rich colourants from various types of polymer matrices as pH indicators in intelligent food packaging systems.

## 2. Materials and Methods

### 2.1. Research Strategy

**Figure 1:**
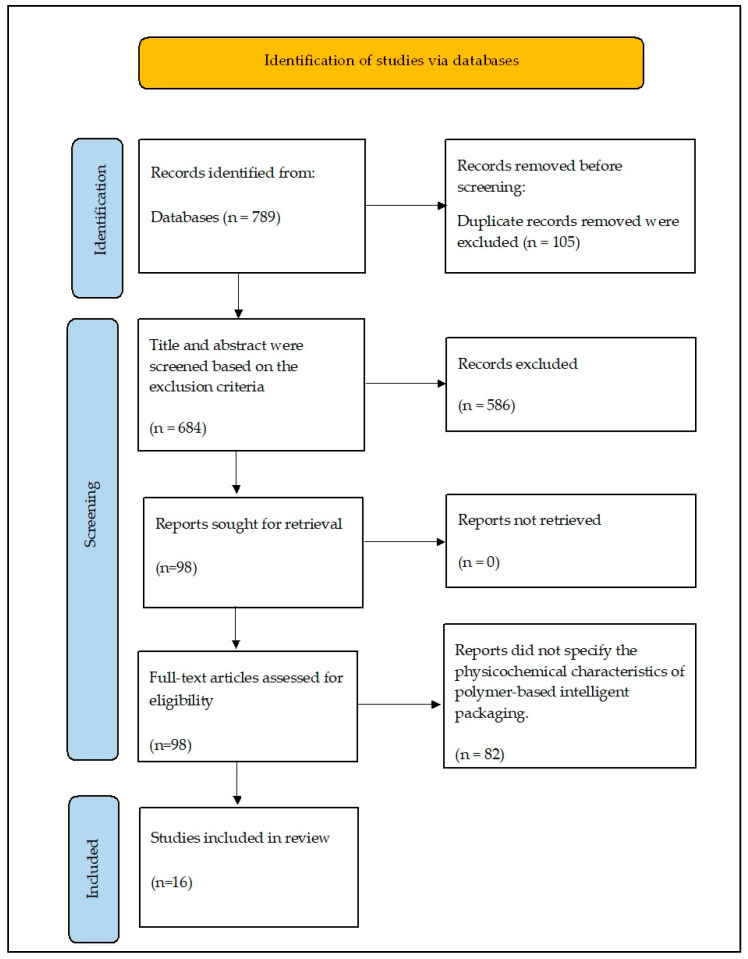
PRISMA flow diagram for article selection process based on the methodology stated [12].

This systematic review paper was written based on the specifications of the Preferred Reporting Items for Systematic Reviews and Meta-Analyses (PRISMA) guidelines [12], as presented in Figure 1, to retrieve articles related to anthocyanins extracted from BPFs (*C. ternatea*) for application in a pH indicator film to monitor foods freshness. Document searching tools provided by Perpustakaan Sultan Abdul Samad (PSAS), the library of the Universiti Putra Malaysia (UPM), Serdang, Malaysia were used. Data were extracted from full-access articles from EBSCOhost, PROQUEST Dissertations and Theses Global, Scopus, SpringerLink, and ScienceDirect using the electronic database search provided by PSAS. Other than that, information was obtained from the general academic search engine Google Scholar. Moreover, in all databases, PICO strategy was used as terms guided by the specific question created, whereby population (P) referred to the research studies on polymer film matrixes for intelligent packaging applications; intervention (I) referred to polymer-carriers immobilised with natural pigment BPF anthocyanin; comparison (C) can be referred to types of polymer film properties; and outcome (O) referred to the application on food products.

### 2.2. Keyword Choices

The keywords chosen were butterfly pea flower; anthocyanin; intelligent packaging; polymer matrix; starch; pH film indicator; freshness indicator; application food; monitoring food. The keywords mentioned above were used as the search strategy. Only the first 100 articles that satisfied all the keywords were reviewed when the search engine displayed too many results. When a lower number of search results was obtained, fewer or more general keywords were used to obtain enough search results.

### 2.3. Inclusion Criteria

The inclusion criteria for this systematic review were determined to screen the eligibility of research articles found in the databases. Studies and articles that fulfilled the following criteria were selected: (a) only articles written in English from research databases were prioritised to save time and to prevent confusion due to mistranslations during the reviewing process; (b) studies that elaborated on the development of intelligent packaging from different types of polymer incorporated with natural pigments as pH indicator films; (c) studies that elaborated on the application of polymer-based strategies for the monitoring of food freshness; (d) articles found in research databases provided by PSAS, UPM, and Google Scholar; (e) articles that were published between the year January 2010 and April 2023, and (f) full-text articles that are accessible.

### 2.4. Exclusion Criteria

Several exclusion criteria were taken into consideration during the reviewing process; (a) articles that only provided abstracts; (b) studies that did not use butterfly pea flowers for the development of intelligent packaging; and (c) articles that did not focus on the current topic.

### 2.5. Study Selection and Data Extraction

The articles searched in the databases were chosen based on their title and the keywords in the title. The abstracts of the shortlisted articles obtained were evaluated while also keeping in mind the inclusion criteria that were set (by N.N.H.). The full article was then gone through to identify whether it satisfied the inclusion criteria. Later, M.R.I.-F. and E.MA. cross-checked the shortlisted articles after reviewing the full texts, and disagreements related to conflicting articles were resolved after a discussion between the four authors (M.R.I.-F., E.M.A., A.R., N.H.Z.A.). The data were collected and tabulated and N.N.H. used self-designed tables to tabulate the relevant data. Table 1 is a summary table for all the selected articles, giving information about the author(s), year of publication, polymer material, the composition of polymer film, food application, and conclusion. Moreover, the information extracted from Table 1 was further divided into two categories; Table 2 provides comprehensive information about the physical and mechanical properties tested on the polymer film. Meanwhile, the applications of the butterfly pea flower films as intelligent packaging are listed in Table 3. A PRISMA flow diagram of the article selection process is shown in Figure 1.

### 2.6. Quality Assessment of the Included Studies

The included research articles were subjected to a quality evaluation step using the Critical Appraisal Skills Programme (CASP) tool [13], which graded each study based on several components, such as the appropriateness of the study design for the research question. Two authors (N.N.H. and M.R.I.-F.) carried this out independently and discussed each item for each study included in this review to reduce the possibility of bias.

**Table 1:** A summary of articles on BPF immobilised in polymer-based intelligent packaging for monitoring the freshness of food.

Author/Year	Polymer Material	Composition of Polymer Film	Food Application	Conclusion
Hashim et al., 2021 [14]	Sugarcane wax/agar (SW/Agr)	Agr/BF/1% SW, Agr/BF/1.5% SW, Agr/BF/2% SW	Shrimp	Agr/BF/2%SW film was chosen as a sensor for shrimps’ freshness due to its overall performance and sensitivity to ammonia vapour.
Wu et al., 2021 [15]	Gellan gum/heat-treated soy protein isolate (G/HSPI)	G/CT, G/HSPI1%/CT, G/HSPI2%/CT	Shrimp	The incorporation of CT extract in G and G/HSPI films successfully reduced the release of CT anthocyanin content.
Ahmad et al., 2020 [16]	Sago (Metroxylon sagu)	SG, SG5/BPF, SG7/BPF, SG10/BPF, SG15/BPF	Chicken	The optimal concentration to formulate the SG film was 5% wt/vol and the surfaces of the films investigated were smooth (complete polymer gelatinisation).
Hidayati et al., 2021 [17]	Chitosan/polyvinyl alcohol (CH/PVA)	CH:PVA (20:80, 40:60, 60:40, and 80:20)	-	CH:PVA (40:60) had the best results for physical and mechanical properties and produced the clearest colour changes with different pH ranges.
Boonsiriwit et al., 2021 [18]	Hydroxypropyl methylcellulose/microcrystalline cellulose biocomposites (HMB)	HMB, 1.0BA-HMB, 1.5BA-HMB, and 2.0BA-HMB	Fish fillet (*Scomber scombrus*)	1.0BA-HMB indicator exhibited the best physical properties; however, 1.5-HMB demonstrated a clear change in the colour response of quality fish (more sensitive).
Sai-Ut et al., 2021 [19]	Gelatin/methylcellulose (G/MC)	G, G/BPE, MC, MC/BPE	-	MC/BPE indicator had improved mechanical and physical properties. Meanwhile, G/BPE showed a clearer response to pH variation.
Koshy et al., 2022 [20]	Soy protein isolate/chitin nanowhisker (SPI/CNW)	SPI, SPI-CNW, SPI-CTE, and SPI-CNW-CTE	-	The addition of CTE to SPI enhanced the mechanical properties. However, the addition of CTE was found to decrease the tensile strength of SPI-CNW film and was found to make the film pH sensitive.
Mary et al., 2020 [21]	Potato starch/nanosized titanium dioxide (S/TiO_2_)	S, S/BPE, S/TiO_2_, S/TiO_2_/BPE	Shrimp	It was observed that the addition of BPE and TiO_2_ could greatly alter the physical properties of the film. The addition of TiO_2_ exhibited changes in colour during the spoilage of shrimp.
Yan et al., 2021 [22]	Chitosan (CH)	CH, CH-BP10%, CH-BP15%, CH-BP20%	Tilapia fish	The incorporation of BP extract increased the thickness, WVP, and mechanical properties of CH-BP films, while reducing their moisture content, swelling ratio, and water contact angle.
Kim et al., 2022 [23]	Gelatine/agar/zinc oxide nanoparticles (Gel/Agar/Zno)	Gel/Agar, Gel/Agar/ZnO, Gel/Agar/BA, Gel/Agar/ZnO/BA	Shrimp	The addition of BA and ZnO significantly increased the UV-blocking properties and surface hydrophobicity without significant changes in the film’s mechanical, thermal stability, and water vapour barrier properties.
Romruen et al., 2022 [24]	Alginate/agar/cellulose nanosphere (CN)	0% CN, 5% CN, 10% CN, 20% CN, and 30% CN.	Shrimp	CN can improve the mechanical properties of smart bilayer films without affecting their chemical properties and proved it is effectively used to monitor shrimp freshness.
Ahmad et al., 2019 [25]	t-carrageenan	Control, t-carrageenan/BPA	Shrimp and durian	The ability of the developed colourimetric pH sensor film from t-carrageenan shows colour changes on shrimp and durian, which provides a simple way to express the quality of food.
Rawdkuen et al., 2020 [26]	Gelatine	Control, Gelatine/BPA	-	The film with BPA extracts in gelatine films showed the highest antioxidant activity, improved water barrier properties, and showed greater pH sensitivity.
Roy et al., 2021 [27]	Carboxymethyl cellulose/agar (CMC/agar)	CMC/agar, CMCagar/ACN, CMC/agar/SKN	-	The incorporation of anthocyanin in CMC/agar-based films improved physical and functional properties without altering the thermal stability.
Sumiasih et al., 2022 [28]	Chitosan/polyvinyl alcohol (CH/PVA)	CH: PVA (20:80, 40:60, 60:40, and 80:20)	Beef	The best formulation was the composition of 20:80 PVA and chitosan 20:80 with the best thickness and total TVBN analysis
Cho et al., 2021 [29]	Corn starch (CS)	CS-BP (9% *v*/*v*, 13% *v*/*v*, 17% *v*/*v*, 20% *v/v*, and 23% *v*/*v*)	Pasteurised milk	The thickness of the films increased with the BP concentration added. Meanwhile, BP solutions incorporating 23% *v*/*v* exhibited the greatest ΔE values.

**Table 2:** pH-indicator composite films based on butterfly pea flower anthocyanin: physical and mechanical changes.

Polymer Film	Main Results after BPFA Incorporation				References
	Physical Properties		Mechanical Properties		
	Thickness	Water Permeability	Tensile Strength	Elongation at Break	
Sugarcane wax and agar matrix	Increase	No significant difference	Low	No significant difference	[13]
Gellan gum and heat-treated soy protein isolate (HSPI)	-	Low	Low	Low	[14]
Chitosan and polyvinyl alcohol (PVA)	No effect	-	High	Low	[16]
Hydroxypropyl methylcellulose biocomposite (HMB)	No effect	-	High	Low	[17]
Gelatine and methylcellulose	No effect	High	Gelatine + BPFA (low) MC + BPFA (high)	Gelatine + BPFA (low) MC + BPFA (high)	[18]
Soy protein isolate (SPI) and chitin nanowhisker (CNW)	No effect	-	Low	Low	[19]
Nanosized TiO_2_	Decrease	Low	-	-	[20]
Chitosan	Increase	High	High	Low	[21]
Zinc oxide nanoparticles (ZnO) + gelatine/agar	Increase	No significant difference	Low	High	[22]
Cellulose nanosphere (CN) and alginate/agar	Increase	No significant difference	High	Low	[23]
Gelatine	No effect	Low	Low	High	[25]
Carboxymethyl cellulose (CMC)/agar-based	No effect	Low	No significant difference	High	[26]

**Table 3:** Application of pH-responsive freshness indicator film of butterfly pea flower in food packaging.

Film Matrix	Foods	Sample Size (g)	Storage (°C)	Visual Colour Change	Final Time (Day)	References
Sugarcane wax and agar matrix	Shrimp	55	25	Deep purple to bluish-green	1.0	[13]
Heat-treated soy protein isolate and gellan gum	Shrimp	-	25	Blue to bluish-green	1.0	[14]
Hydroxypropyl methylcellulose biocomposite (HMB)	Mackerel fish	200	4	Deep purple to violet	6.0	[17]
Nanosized TiO_2_	Prawn	20	4	Pink to green	6.0	[20]
Chitosan	Tilapia fish	-	4	Purple-blue to dark green	6.0	[21]
t-carrageenan	Durian and shrimp	-	28	Shrimp: deep blue to greenish-blue Durian: deep blue to dark purple	Shrimp: 0.5 h Durian: 4.0	[24]
Chitosan and polyvinyl alcohol (PVA)	Beef	60	25	Blue to bluish-green	1.0	[27]
Corn starch	Pasteurised milk	250 (mL)	25	Deep blue to light blue	3.0	[28]

## 3. Results and Discussion

### 3.1. Study Characteristics

The PRISMA flow diagram in Figure 1 describes the entire screening process of articles in the review, where we determined the eligibility of research articles found in the database. Firstly, the timeline was set to 13 years, so articles that were published between 2010 and 2023 were included in the review process. In terms of content, the selected research articles were identified and discussed for the studies that focused on the development and investigated the ability of different types of polymer matrix films to be incorporated with butterfly pea flower extract on intelligent packaging specifically as a freshness/pH indicator. Furthermore, the application of butterfly pea flower anthocyanin as a polymer-based pH film indicator was included in the review process where the effectiveness of the pH indicator was in monitoring the freshness of various types of food (e.g., seafood, poultry, milk) stored at room temperature or chiller conditions at 4 °C. The characteristics of all the research involved (summary) are displayed in a data extraction table in Table 1.

### 3.2. Butterfly Pea Flower (Clitoria ternatea L.)

#### 3.2.1. Plant Morphology

*Clitoria ternatea* L. belongs to the Fabaceae family. It is also called the Butterfly Pea, Blue Pea, Asian Pigeonwings, and *Telang* (in the Malay language). It is a climbing flowery plant creeping towards other plants in competition for sunlight. The flowers are big and solitary and the most striking feature is the colour of the flowers, which have a vivid deep blue to mauve colour [30] with yellow in the middle and a white spot at the edge. The colours in the flower petals are due to the presence of anthocyanin content such as delphinidins.

**Figure 2:**
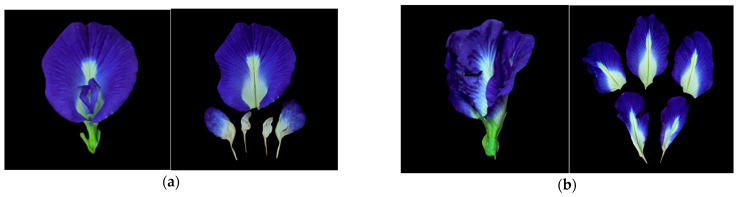
Variation structure of the butterfly pea flower: (**a**) normal corollas; (**b**) multiple layered corollas.

According to Suarna and Wijaya [4], BPFs have two different kinds of corollas (the group of petals in the flower) and stamen: (Figure 2a) a normal corolla has four petals, where two petals at the lateral area called wings, and other two petals at the posterior area called carina with diadelphous stamen. On the other hand, a multiple-layer corolla (Figure 2b), which has five petals of corollas, consists of one petal (the biggest) at the anterior area with 10 solitary stamens. Havananda and Luengwilai [31] stated that the reproduction of BPFs is conducted through fruit seeds, and it quickly shows excellent regrowth after cutting or grazing and produces a high yield of blooming flowers. BPFs flourish when lightly grazed throughout the wet season.

#### 3.2.2. Plant Pigment (Anthocyanin)

Anthocyanins (Anthos is flower and kyanos is blue in Greek) are polar compounds that are water-soluble pigments, belonging to the flavonoids compound, where they are a subclass of the polyphenol family [32]. They provide a colour spectrum in the range of red, blue, and purple, as seen in the higher plants. They are frequently found in flowers and fruits. However, they can also be found in other plants, including vegetables, legumes, and cereals. According to Khoo et al. [33], anthocyanins are glycosides of anthocyanidins, which are often found in a variety of plant species such as cyanidin, delphinidin, pelargonidin, peonidin, petunidin, and malvidin. Moreover, BPFs have medicinal properties. The extracts of anthocyanin have been used over the years as a potential antioxidant, antimicrobial, anti-inflammatory, and antidiabetic activity [34] and, more recently, due to studies indicating anti-cancer properties [35].

In BPFs, a high abundance of polyacylated anthocyanins in the flower is responsible for the plant pigment’s stable blue colour [36]. Anthocyanins that are commonly found in BPFs are ternatin and delphinidin [37]. A study by Ahmad et al. [25] found that 12 different compounds of anthocyanin were successfully detected in BPFs where ternatins were the largest anthocyanin groups. Chemically, ternatins are blue and they consist of anthocyanidin or aglycon and sugars with the presence of a third component, which is delphinidin. The primary anthocyanin responsible for this flower’s intense blue to purple colour is delphinidin [38]. Jeyaraj et al. [34] reported that the stable polyacylated derivative of delphinidin 3,3′,5′-triglucosides called ternatin is found in the petals of BPFs. It has 3′,5′-side chains with alternate D-glucose and p-coumaric acid units at R and R1. Thus, delphinidin-3,3′,5′-triglucoside is the fundamental structure of all ternatins in BPFs.

Azima et al. [39] reported that when compared with other natural colourants, BPF anthocyanin has a more intense, vibrant, and saturated colour due to significantly higher colour density and chroma values. Furthermore, it was found that cyanidin-3-(p-coumaroyl-glucoside) was the most prevalent anthocyanin present based on its intensity [1] and was identified as the anthocyanin in the aqueous extract of BPFs’ blue petals.

#### 3.2.3. pH Sensitivity of Anthocyanin

**Figure 3:**
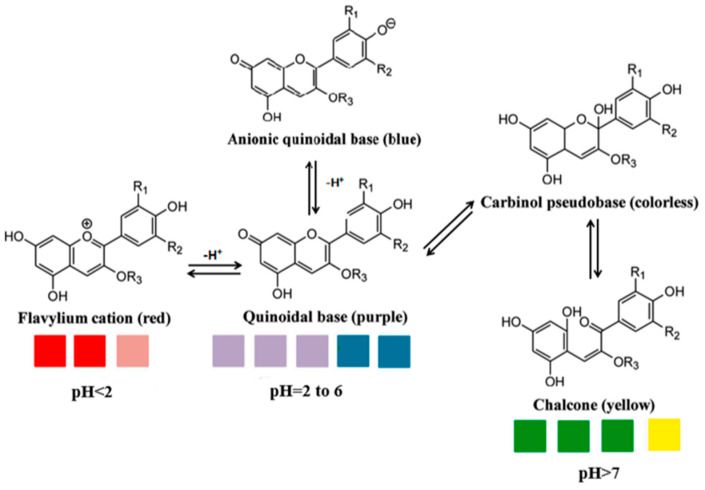
The main forms of anthocyanins at varying pH [6].

Anthocyanins, water-soluble compounds that can create a broad range of colours (red, pink, purple, and blue), are commonly extracted from flowers, vegetables, cereals, and fruits. Additionally, Balbinot-Alfaro et al. [40] reported that anthocyanins can be detected in different colours and chemical forms depending on the pH of the solution, and this could be used to track food products over their shelf-life and, eventually, monitor food quality criteria. The source, composition, and structure of anthocyanins are related to the reversible colour characteristics of anthocyanin-rich plants [6]. The ability of anthocyanin to change colour is a unique characteristic. Four different coloured anthocyanin forms can alternately change based on the pH of the solution ranging from 1 to 13, as shown in Figure 3.

Based on Figure 3, the anthocyanin-rich extract’s colour changes can be seen when the pH rises gradually from the acidic to the alkaline area. Because of the bathochromic and hyperchromic characteristics [41], the colour of the anthocyanin-rich extract is at pH values pH < 2; where it is at very acidic pH values, the formation of the red flavylium ion (AH+) is favoured. This species is fully protonated and has a delocalised positive charge across the chromophore. With a slight increase in pH, the first deprotonation occurs where kinetic and thermodynamic competition happens between the hydration reaction of the flavylium cation and the proton transfer reactions related to its acidic hydroxyl groups, thus converting into the neutral quinoid base (purple). The colour changes towards a red to purple quinoidal base. At the pH values between six and seven, the quinoid base (purple colour) is deprotonated further, forming the anionic quinonoid base (blue) with a negative delocalised charge, and the colour changes from purple to blue [42]. As the pH is raised from acidic conditions to slightly acidic or nearly neutral circumstances, this blue colour will appear.

The stability of anthocyanins gradually declines as pH increases (pH > 7), where it is dependent on their substituent groups and will generate–a green-light yellow colour as a result of isomerisation into chalcone formation via water catalysed-tautomerisation [43]. It is well-known that anthocyanin properties, including colour expression, are highly influenced by anthocyanin structure and pH. Due to these abilities, anthocyanins extracted from BPFs could potentially act as naturally derived pH dyes for colourimetric indicators in the monitoring of seafood and poultry freshness since they provide significant changes in the colour spectra with the changes in pH, and they are a non-toxic compound and give lower risk to the consumer. Therefore, the pH indicator sensitivity of BPF anthocyanins may be beneficial to be facilitated in intelligent packaging systems.

### 3.3. Anthocyanin Extraction from BPFs

**Figure 4:**
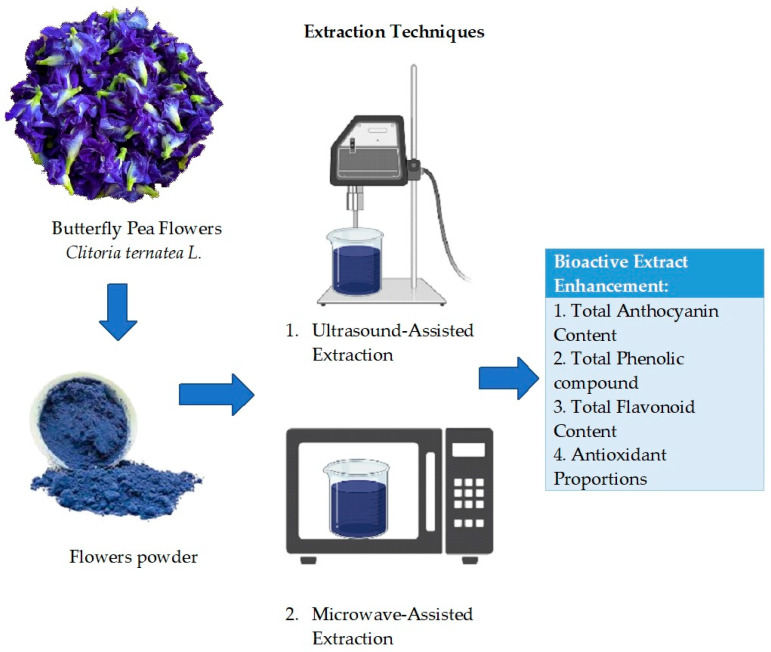
The non-conventional extraction processes of BPF.

Extraction is the most important step to isolate polyphenols and anthocyanin as natural colourants from plant sources. Various factors affect the overall extractable yield of anthocyanin in *Clitorria ternatea* that are included in the stability/sensitivity of BPFs such as temperature, pH, light, presence of enzymes, oxygen, metal ions, sulphur dioxide, and phenolic acids [44]. The most important factor to extract anthocyanin from plant sources is the selection of solvent, extraction time and temperature, and the ratio of the extractable substrate (flower) to solvent ratio [39], which will affect the anthocyanin content in the BPF. Traditionally, the direct addition of a powdered form of the dried BPF is a common procedure for the extraction process as shown in Figure 4. Prior to extraction, plant materials are prepared by size reduction using a grinder on either fresh [16] or dried samples [45] to increase the surface area for mixing with solvent. On the other hand, extraction can be divided into two methods, which are either conventional or non-conventional extraction methods to extract anthocyanins from the flower. Therefore, for this review, only extraction techniques that can save time and cost and produce a high extraction yield of anthocyanin from BPF anthocyanin will be discussed.

#### 3.3.1. Extraction Solvent

Plant extraction involves separating the desired plant material components from inactive or undesirable compounds [39], mainly with the help of a liquid solvent. The alcohol-extracted anthocyanins exhibit greater functionality and higher effectiveness in their research on solvent extraction parameters on the quality of BPF extraction [6]. The expansion of the plant matrix by the water increased interactions between the surface area of the plant material and the solvent, and it increases the alcohol’s ability to be extracted from the plant material [38]. Examples of alcohol that may be used in anthocyanin extraction are ethanol, methanol, acetone, or mixed solvent with water. The best solvent for natural compounds used for both food and natural medicine is ethanol, which is safe to be consumed by humans. However, using methanol should not be used as a solvent because it is a toxic, water-miscible, and volatile corrosive alcohol.

Tena and Asuero [46] stated that a high amount of ethanol in the solvents promotes the extraction of bioactive chemicals from plant materials, such as anthocyanin in flavanol groups. This is because the presence of aromatic groups and glycosyl residues in anthocyanins causes molecules to dissolve more effectively in a polar solvent which is easily absorbed by cell membranes on the surface of tissue particles in flower petals [41]. In addition, they discovered that using water and ethanol (20–70% *v*/*v*) together as a solvent improved the amounts of monomeric anthocyanins compared with using either water or ethanol alone [46]. Jeyaraj et al. [34] found that 50% ethanol (50–50% *v*/*v*) was discovered to be the best solvent for BPF extraction with 57.3% extracted yield and a 5.1 mg/g of total anthocyanin content compared with other types of solvent. Therefore, the type of solvent extraction process, specifically, alcohol mixed into water, affects the levels of anthocyanin.

#### 3.3.2. Conventional and Non-Conventional Extraction Methods

The conventional extraction method is a traditional method that has been used since the 1970s for the extraction of BPF. Soxhlet extraction, maceration, and hydro distillation are among the conventional methods that usually involve the use of different solvents with heat and mixing. These methods are effective, but they consume more solvents, time, and thermal energy and are also associated with several disadvantages [47]. Therefore, to overcome the shortcomings of conventional solvent extraction and to increase extraction efficiency, several non-conventional extraction techniques were explored. Non-conventional extraction methods consist of ultrasound-assisted extraction [45], enzyme-assisted extraction, supercritical fluid extraction, microwave-assisted extraction, and pressurised liquid extraction [48]. These techniques are emerging, rapid, eco-friendly, and highly efficient over conventional extraction methods. According to Vidana et al. [36], ultrasound- and microwave-assisted extraction are the best methods to be applied for the extraction of anthocyanin from BPFs. As for now, however, among the non-conventional extraction methods, only ultrasound- and microwave-assisted extraction have been conducted to extract phytochemicals and it has been shown to improve the bioactive compound on *C. ternatea* flowers as shown in the processes in Figure 4.

#### 3.3.3. Ultrasonic-Assisted Extraction (UAE)

Ultrasonic-assisted extraction (UAE) is a renewable and eco-friendly technology that uses acoustic cavitation to cause molecular movement of the solvent and sample, resulting in the breakdown of plant cell walls and membranes, and thus facilitating their movement to the surrounding solvent [49]. Husin et al. [45] conducted a study using UAE to extract anthocyanin from BPF as a visual indicator for monitoring meat freshness. Apart from that, comparisons were performed between UAE and conventional extraction methods on the extraction of anthocyanins from BPF. It was found that using UAE showed 246.48% better anthocyanin yield with 1.126 mg/g of total anthocyanin content [50].

The UAE procedure can be carried out with a bath ultrasonic (BUE) or probe ultrasonic (PUE). According to Kumar et al. [51], BUE is more practical and cost-effective. However, the energy generated is not evenly spread throughout the bath, which lowers the extraction’s effectiveness. Consequently, it is less reproducible than PUE. PUE consists of a probe connected to a transducer, which is applied directly to the sample. The probe is directly immersed in the extraction and disperses the ultrasound in the media with the least amount of energy loss. PUE delivers a more intense ultrasonic sound than the bath device. However, there is not any research showing a comparison between BUE and PUE for BPF extraction. In PUE, the extraction process consists of four steps [52]: (1) when ultrasound waves pass through the solvent, cavitation bubbles are generated near the outermost layer of the plant matrix; (2) the bubbles erupt, releasing a microjet of higher temperature and pressure; (3) the plant matrix surface is ruptured, creating direct contact between the cell membrane and the solvent; (4) the solvent solubilises and transports the intracellular components, making the anthocyanin of the plant during extract more efficient. The ultrasonication process has an impact on the base fluid’s agglomerates and nanoparticle sizes [53]. Therefore, probe ultrasonication is frequently chosen to extract bioactive compounds compared with the use of bath ultrasonication.

#### 3.3.4. Microwave-Assisted Extraction (MAE)

Microwave-assisted extraction (MAE) is known as a green technology that uses microwave energy to facilitate the segregation of analytes which are high recovery and solvent blends from the plant matrix into the solvent [54]. The major factor that determines the efficiency of microwave energy is the choice of the solid-to-solvent ratio, plant matrix properties, irradiation time, and irradiated microwave power [55]. Theoretically, the microwave induces dipole rotation of the molecules, which makes electromagnetic energy convert to heat and disrupts the hydrogen bonding, thereby increasing the migration of dissolved ions and promoting solvent penetration into the plant matrix [56]. Various studies have been performed using the microwave-assisted technique to extract natural colourants from BPFs. Gamage and Choo [57] compared the extraction efficiency of natural BPF colourant (anthocyanins) using conventional extraction with water and heating of MAE. MAE was more efficient (9.61 mg/g) than conventional extraction (5.22 mg/g), having a higher total anthocyanin content.

According to the study by Marsin et al. [58], the anthocyanin content of the BPF extract had a short extract time, within 1 min, and obtained 0.541 mg/g of anthocyanin content. This is because Farzaneh and Carvalho [59] mentioned that increasing the time of microwave irradiation might degrade the anthocyanin content and reduce the effectiveness of extracting all of the anthocyanin yields in the plant. In contrast, Romero-díe et al. [60] stated that a longer exposure time to MAE improved extraction yield and simultaneously enhanced processing temperature, which may have contributed to the higher yield of anthocyanin extraction; this study was supported by reference [61], where the anthocyanin content of the 15 min MAE was 0.457 mg/g. Therefore, MAE is considered an efficient approach to extracting valuable active compounds from plant materials, maintaining their natural colourant, and being time efficient and easy to handle.

### 3.4. Intelligent Packaging

An intelligent packaging system consists of inexpensive components and compact labels or tags that can collect, store, and transmit data about the features and characteristics of packaged food [62]. Intelligent packaging can monitor, capture, and record changes in the product or its external environment and allows it to deliver information to the consumer as an extension of the communication function of conventional food packaging [63,64]. It is crucial to note that intelligent packaging and active packaging are two different things, but some packaging systems may be classified as both. According to Fang et al. [65], active packaging enhances the protection function of conventional packaging, and it is designed to contain a component that enables the release or absorption of substances (e.g., the release of an antimicrobial or antioxidant) into or from the packaged food or the environment surrounding the food to increase shelf-life and food safety and quality. Active packaging and intelligent packaging are compatible. Both packaging systems can work synergistically to achieve the quality of smart packaging. Smart packaging also offers a comprehensive packaging solution that both actively responds to and intelligently analyses changes in the product or environment [66].

#### 3.4.1. Time-Temperature Indicators

Temperature is typically the most important environmental factor determining food spoilage and deterioration [67]. The term “time-temperature indicator or integrator” (TTI) refers to a straightforward, reasonably priced device fastened to shipping containers or individual consumer packages that can display measurably time-dependent changes reflecting the entire or a portion of a food product’s temperature history [65]. The main mechanisms of action include enzymatic reactions, polymerisation, and chemical dispersion [66]. Therefore, TTI is useful; it can inform about temperature abuse and could be used as an indirect shelf-life indicator for perishable food products.

#### 3.4.2. Integrity Indicators

Integrity indicators are one of the components of intelligent packaging that function as time indicators and reveal how long a product has been opened. They are also called seal and leak indicators or gas indicators. The application of this intelligent packaging is that foods are packaged with the essential gas composition for storage [66]. This is because the gas composition in the package frequently changes because of the water activity of the food product, leaks, the nature of the package, and external environmental factors [68]. Realini and Marcos [63] stated that when a seal is broken, an integrity indicator activates the label now that it is being consumed, initiates a timer and colour changes to happen throughout time. The functionality of most devices is based on redox dyes, a reducing compound, and an alkaline component [69].

#### 3.4.3. Freshness Indicators

Freshness indicators or pH indicators are such devices that can directly be used to provide an estimate of the remaining shelf-life of perishable products [68]. Most of the pH or freshness indicators cause an alteration in colour in the sensor due to the presence of volatile amine compounds and increasing pH during the spoilage of food products. Horan [70] reported that biogenic amines (putrescine, cadaverine, histamine) are created when proteins in perishable food are broken down into amino acids and then those amino acids are enzymatically decarboxylated. Therefore, the production of biogenic amines from muscle foods can give a direct signal to a pH indicator (colour change), which acts as an indicator of food deterioration.

Bromophenol blue pH dye is the most applied in the muscle food packaging industry to monitor freshness from the production of carbon dioxide caused by microbial growth from protein degradation [69]. Additionally, high carbon dioxide levels cause pH dyes to react and change colour. However, bromophenol blue is a synthetic dye and this synthetic colour is prone to cause problems; it can be hazardous, especially if it is to be used for food purposes. Synthetic dyes such as methyl red and bromocresol green might be toxic (harmful) to humans, and can cause lung disease and skin infection [71]. Therefore, synthetic dyes were replaced with anthocyanin, which is a water-soluble natural pigment (non-toxic) from plant material. There are numerous types of freshness/pH indicators that have been developed recently by immobilising anthocyanin. Apart from that, anthocyanins are responsible for the blue, red, or purple colour based on the anthocyanin’s source and composition, where it can visibly change colour when the pH of the surrounding area changes.

### 3.5. Application of BPF Anthocyanin (BPFA) as a Polymer-Based pH Film Indicator

**Figure 5:**
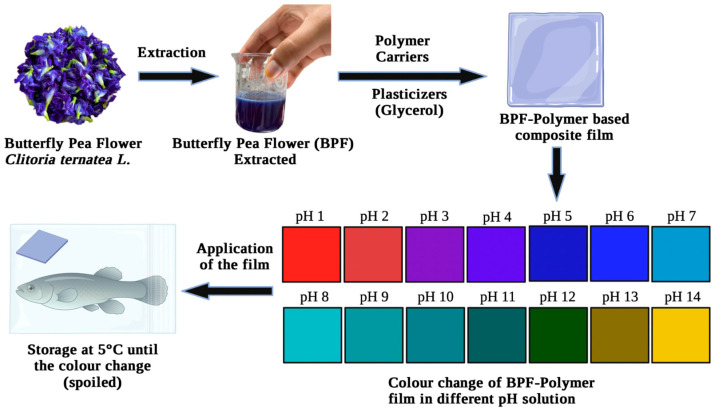
Fabrication of BPF–polymer-based pH indicator film.

Studies have developed intelligent packaging with natural pH indicators (BPF) due to their inexpensive cost, safety in contacting food materials, and ability to monitor with the naked eye the pH changes caused by food deterioration. The films can be developed using many types of polymer-carriers with different properties and characteristics. Figure 5 illustrates the fabrication of the BPF–polymer-based film and the colour changes of the film in different pH solutions (pH 1–pH 14).

#### 3.5.1. Effect of BPFA on the Physical Properties of Films

##### Thickness

The physicochemical, light transmission, and barrier properties (water, gas) of the produced polymer matrix are all influenced by the thickness of packing films, making them a crucial factor [6]. The polymer matrix’s composition and the dispersibility of anthocyanin have a significant impact on the pH indicator film. Several studies reported that low concentrations of anthocyanin do not significantly alter the thickness of the films because anthocyanins can distribute evenly throughout the film matrix [26]. Hidayati et al. [17] indicated that the thickness of the film is not significantly influenced by the immobilisation of BPFAs; this is because of the fixed BPFA concentration (ratio), and proper solubility of BPFAs mixed with the chitosan: polyvinyl alcohol (CH: PVA) composite matrix. Similarly, Sai-Ut [19] stated that there is no significant difference in the thickness of the gelatine and methylcellulose films’ thickness. This might be caused by the extract’s small volume and low moisture content in film compositions.

On the other hand, the stability of the polymer film and the thickness of the film were unaffected by increasing anthocyanin content. On this matter, the thickness of pH-colourimetric hydroxypropyl methylcellulose (HM) films recorded no significant difference with the addition of BPF anthocyanin content [18]. In contrast, Yan et al. [22] stated that increases in the amount of BPFA-loaded chitosan films have significantly increased compared with the control films without BPFA. In line with these findings, the incorporation of BPFA resulted in a higher thickness value of colourimetric corn-starch-based films, and sago-starch-based films recorded the lowest value [17].

Following these studies, based on the composition of the polymer matrix and the amount of BPFA properties, it can be concluded that the addition of BPFAs had a variety of effects on the thickness (thicker or thinner) of pH indicator intelligent films. Table 2 shows the type of polymer used and their physical and mechanical properties when incorporated with BPFA.

##### Water Vapour Permeability

Water vapour permeability (WVP) is an important aspect of evaluating the effectiveness of polymeric materials used in packaging films for preventing moisture transfer (permeability) attributes into food packaging structures to act as barrier properties and is considered an important criterion in monitoring food quality and safety [72]. The prevention and minimising of oxygen and moisture transfer between the packaged food and the external environment are crucial factors of food packaging systems to prevent food spoilage from happening quickly [26]. Yan et al. [21] stated that the WVP of the film matrix was significantly higher due to the hydrophilicity of chitosan and that with the increasing amount of BPFA, the wettability of the film surface was enhanced. Polyphenolic molecules of BPFA possibly caused low intermolecular interactions in the film network. Thus, the hydroxyl groups of BPFA molecules might cooperate with water and intervene with the network formation of hydrogen bonds.

Moreover, Sai-Ut et al. [18] stated that there was an increased WVP in gelatine and methylcellulose composite films when they were infused with BPFA. The value of WVP can be influenced by the incorporation of bioactive compounds. On the other hand, Wu et al. [15] reported that the incorporation of BPFAs and gellan gum/heat-treated soy protein isolate (G/HSPI) matrix films significantly decreased the WVP value. The research reported that these changes may be linked to the addition of anthocyanin compounds (polyphenols) into polysaccharide- (gellan gum) or protein-based (soy protein isolated) films, which resulted in the interactions between the polymer-based matrix film and BPFA due to the hydrogen bonds and noncovalent hydrophobic interactions were formed when polyphenol (anthocyanin) were added to polysaccharide- or protein-based films [73], Moreover, the BPFA structure’s aromatic rings have an impact on the development of a stronger microstructure link, which lowers the polymer chain’s binding affinity to water molecules.

Mary et al. [20] also reported that the WVP value of starch with titanium dioxide (TiO_2_) was decreased with the addition of BPFA. The probable cause for this result was the restriction of water vapour pathways in the polymer film matrix by TiO_2_ nanoparticles. Moreover, titanium oxide particles bind with the starch hydroxyl group to form a complex network that inhibits the hydrophilic groups’ capability for the absorption of water vapour. However, Hashim et al. [14] stated that the incorporation of BPFA into sugarcane wax/agar matrix films does not change the WVP value of the films. Many variables affect the permeability of the film, such as how the concentration of plasticiser should be compatible with the film-forming polymer [74], the different proportions of biopolymer during the development of the film, as well as the drying and storage time effect [72].

#### 3.5.2. Effect of BPFA on the Mechanical Properties of Films

Food products’ sustainability and integrity could be ensured by matrix films with sufficient mechanical strength [6]. There are two major mechanical criteria for judging the durability and flexibility of packaging films: tensile strength (TS) and elongation at break (EAB). According to Yong et al. [5], TS is defined as the maximum tolerance of composite films against the applied stress while being pulled or stretched before breaking occurs. Moreover, EAB is defined as the maximum capability of composite films to maintain alterations in the length and shape of the films deprived of any crack formation. Generally, the mechanical properties (TS and EAB) of BPFA-rich films can be affected by various factors. In this regard, Koshy et al. [19] reported that the TS of the BPFA mixed with soy protein isolates/chitin nanowhisker (SPI/CNW) composite films decreased with the addition of BPFA. This might be due to the occurrence of several interactions between BPFA and the composite matrix due to the plasticisation impact of BPFA, which breaks the compact, stiff structure between SPI and CNW by promoting molecular mobility of the polymer chains and destroying the hydrogen connection between SPI and CNW, despite the EAB value showing a significant increase. This could be explained by the extract’s phenols and flavonoids operating where they are repulsive forces to each other, decreasing the attraction between the chains of proteins.

Nevertheless, in research by Wu et al. [14], the addition of BPFA in the gellan gum/heat-treated soy protein isolate G/HSPIs composite significantly decreased the values in both mechanical TS and EAB. The presence of the anthocyanin-rich extract may weaken the intermolecular interaction and discontinuous microstructure of gellan gum and heat-treated soy protein isolate film. In contrast, Yan et al. [21] stated that up to 20% concentration of BPF anthocyanin pigment was added to cationic chitosan matrix films, both TS and EAB values were significantly increased because of the many hydroxyl groups in anthocyanin that create hydrogen bonds and result in significant interfacial adhesion between the polymer and BPFA extract. The additional anthocyanin compound may increase the volume of molecules that can move freely, enhancing the flexibility of the composite film.

Moreover, the difference in anthocyanin source could have a significant impact on the mechanical properties of the polymer-based matrix films loaded with BPFAs. For instance, in Boonsiriwit et al.’s [17] work, the hydroxypropyl methylcellulose, HMB-based intelligent packaging films contained an increasing amount of anthocyanins demonstrated an increasing TS; however, the tensile strength of the highest BPFA-HMB was not significantly different from that of the control (without BPFA). The changes in TS can be clarified based on the strength of the electrostatic contacts between anthocyanin molecules and the HMB composite, as well as the intermolecular interactions [74], while a lower EAB was found with a significant increase in the BPFA concentration. This was due to the high concentration of phenolic chemicals in BPFA, which restricted polymer chain motion and prevented chain–chain connections of polymeric backbones; this was attributed to the interactions of BPFA with the HMB matrix, which decreased the flexibility of the films. Overall, the type of polymer used, co-film forming elements (plasticiser) and BPFA concentration could influence the mechanical properties of BPFA-loaded matrix films [69].

### 3.6. Evaluation of BPFA pH Indicator Potential Tested on Food

**Figure 6:**
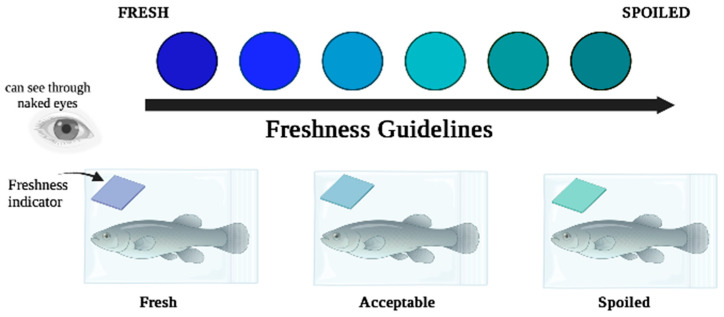
Typical freshness indicator system applied to food.

Natural biopolymers, particularly those based on polysaccharides and proteins, have been utilised extensively to create colourimetric intelligent packaging films because of their biodegradability, nontoxicity, safety, stability, and good film-forming capabilities [6]. Figure 6 illustrates the typical freshness indicator system and demonstrates the colour changes when the pH increases from blue (fresh) to green (spoiled) inside the food packaging, while Table 3 shows the types of film matrices used, foods and their storage conditions, and colour changes observed in the films.

Ahmad et al. [25] reported good performance of an edible BPFA pH indicator immobilized in t-carrageenan, as it was employed to track the pH alterations of durian at room temperature. In addition, the pH-sensitive indicator can be used to observe pasteurised milk, as it can easily distinguish between fresh and deteriorated milk using colour detection and will show a deep blue to the light blue colour range [29]. Boonsiriwit [17] developed a pH-colourimetric indicator film using a hydroxypropyl methylcellulose biocomposite and BPFA. It was observed that the colour of the anthocyanin compound on the indicator film changed from deep purple to violet on mackerel fish storage. Approximately 1.5 g of BPFA indicator exhibited the most efficient indicator in terms of NH_3_ reactivity. The 1.5 BPFA-HMB indicator clearly changed colour in response to variations in mackerel fish quality.

Chitosan is the most common natural biopolymer that has been used in the fabrication of smart films with BPFA. Yan et al. [20] monitored tilapia fish deterioration using chitosan film immobilised with BPFA. According to the research, colour alterations in the film were seen as a result of the fish sample’s various storage conditions at room temperature, and it was observed that no colour change was seen on the freshness indicator film after day two. However, after day four of storage, the colour began to shift to green, which showed initial spoilage and pH increase. After day six of storage, the colour completely turned to dark green, indicating that the tilapia had undergone complete spoilage. Sumiasih [27] developed an intelligent pH indicator film using chitosan and PVA and evaluated a BPFA to monitor beef spoilage. As a result of the film’s high sensitivity to pH changes, the results showed that the indicator film’s initial blue colour drastically altered to a greenish colour within 24 h at room temperature storage, which may undergo a decomposition process. Generally, as the seafood and poultry deteriorated, it produced an unpleasant aroma from the formation of volatile alkaline compounds such as ammonia, dimethylamine (DMA), and trimethylamine (TMA) from enzymatic reactions [75]. Volatile alkaline was produced as bacteria degraded proteins into amino acids [70] and oxidation of unsaturated fatty acids in the muscle food body occurred [76]. The BPF’s anthocyanin characteristics are sensitive to variations in acidity. Therefore, the pH change from acidic to alkaline can be detected by the anthocyanin in the BPF extract changing colour.

## 4. Conclusions

Recently, the immobilisation of butterfly pea flowers’ anthocyanin (BPFAs) into polymer-based films has provided advancements for intelligent food packaging application systems. Incorporating BPFA into the starch composite of packaging films often causes different changes in their physicochemical properties, mainly due to interactions between natural polymer-carriers and hydroxyl groups in anthocyanins. Furthermore, the BPF is a plant that is easy to obtain, is not seasonal, and has potential features such as antioxidant capabilities in addition to pH sensors in food packaging systems to prolong shelf-life. To conclude, the BPF has a promising future for intelligent polymeric films using BPFAs due to their multifunctional utilisation such as monitoring and improving food products and consumer safety.

## Data Availability

Not applicable.
